# Supplementary material for: Evaluation of circulating plasma proteins in breast cancer using Mendelian randomisation
Source: Nat Commun. 2023 Nov 24;14:7680. doi: 10.1038/s41467-023-43485-8 (PMC10667261; doi:10.1038/s41467-023-43485-8)
Supplement: Supplementary file 1 — Supplementary Information [file 41467_2023_43485_MOESM1_ESM.pdf]

Supplementary figures

Supplementary figure 1. Distributions of the proportions of proteins above the protein limit of detection per Olink Explore panel

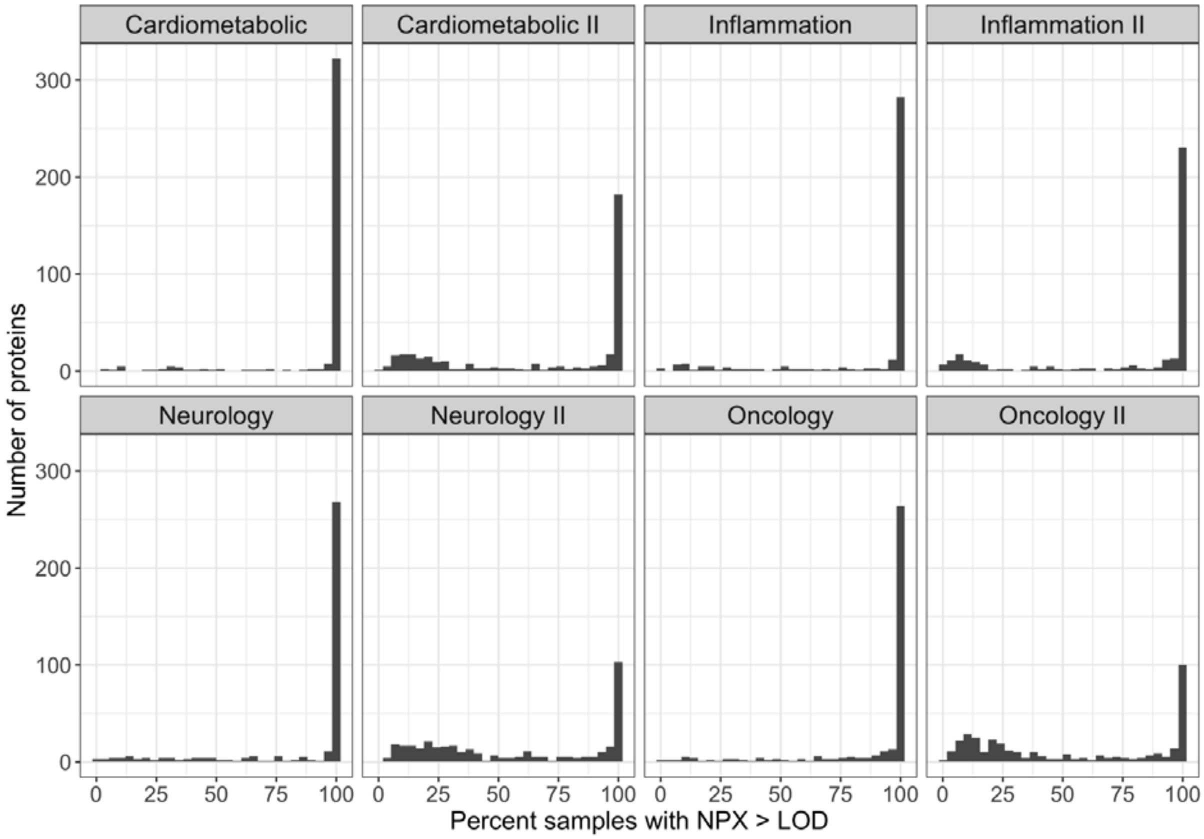

Supplementary figure 1: Histograms showing the percent of samples with NPX above the limit of detection (x-axis) per protein. The number of proteins in each bin are indicated on the y-axis. Each figure-panel corresponds to one of the 8 panels into which the ~3K proteins are grouped. LOD stands for limit of detection.

Supplementary figure 2. NPX distributions per panel

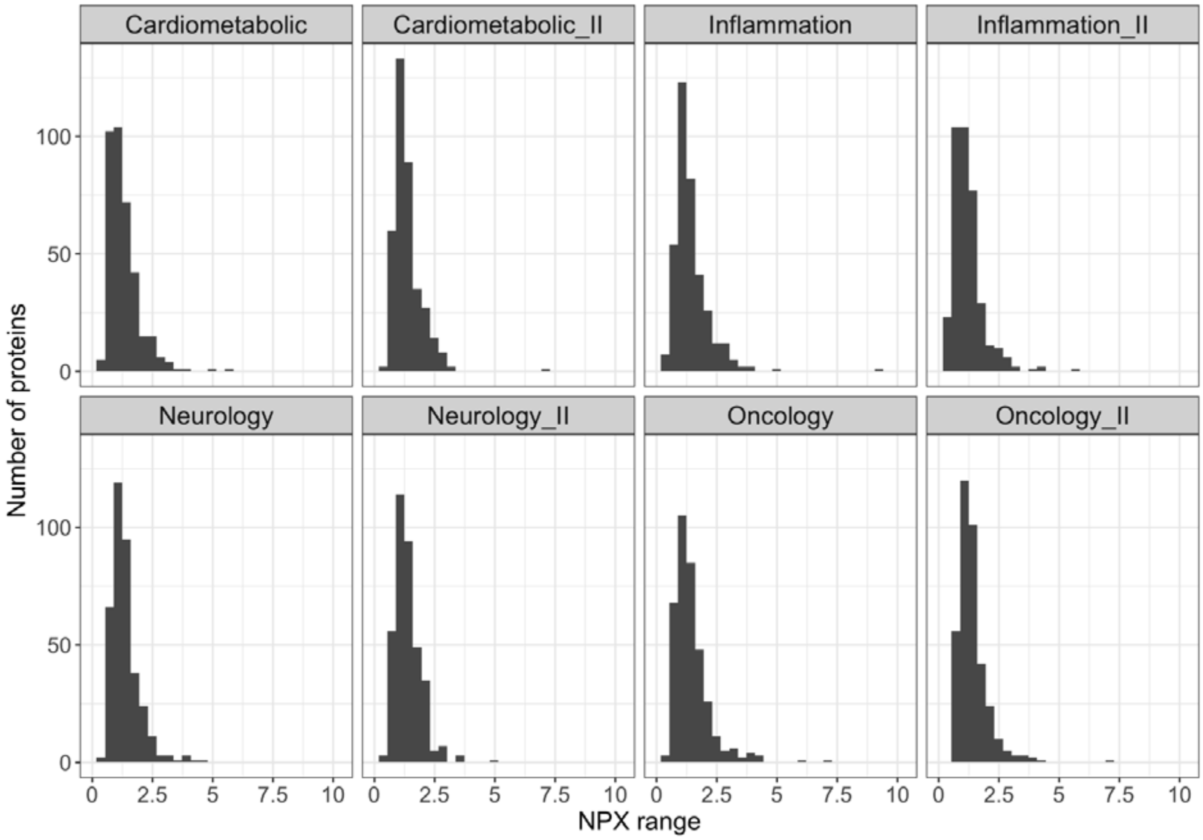

Supplementary figure 2: Histograms showing the range of NPX values (x-axis), defined as the 90th percentile - the 10th percentile, per protein. The number of proteins in each bin are indicated on the y-axis. Each figure-panel corresponds to one of the 8 panels into which the ~3K proteins are grouped. The ranges per protein (calculated as the 90th percentile - the 10th percentile to avoid outlier effects) varied between 0.17NPX and 9.27NPX, with an average of 1.36NPX, corresponding to a 2^1.36 ~2.6-fold difference in protein abundance between 10th and 90th percentiles (supplementary figure 2).

Supplementary figure 3. Estimated protein abundance for the subset of proteins that overlap with Olink Explore, using data from the Human plasma peptide atlas.

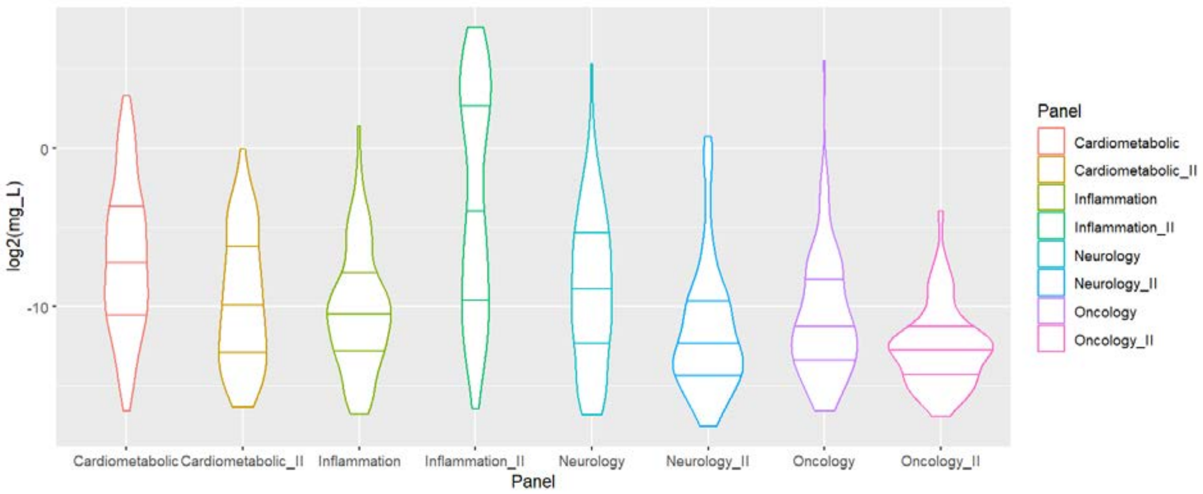

Supplementary figure 3: Violin plots showing the plasma protein abundance for proteins that overlap with the Olink Explore PEA assay. The concentration data were based on community data using mass spectrometry. The predicted values originate from the 2021 Human Plasma PeptideAtlas Build (https://peptideatlas.org) and were obtained from the Human Protein Atlas project (www.proteinatlas.org).

Supplementary figure 4. Association of proteins with incidence breast cancer.

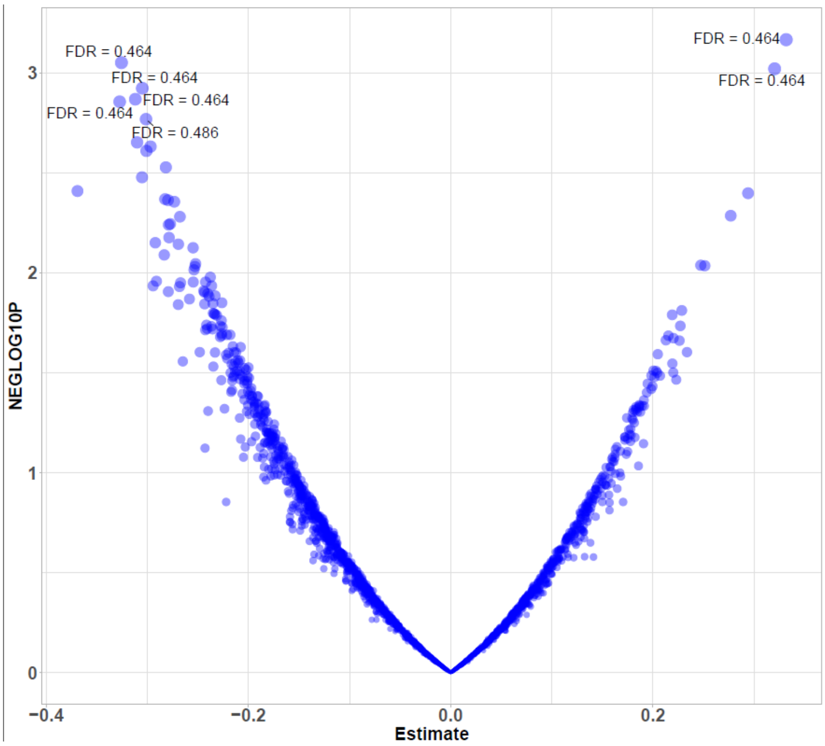

Supplementary figure 4: The comparison of plasma protein levels between all breast cancer cases and controls, using a linear generalised model adjusted for age with 5 % false-discovery rate adjustment for multiple testing revealed no proteins significantly different between incident breast cancer and controls.

Supplementary figure 5. Estimation of the number of independent single nucleotide polymorphisms per cis-region

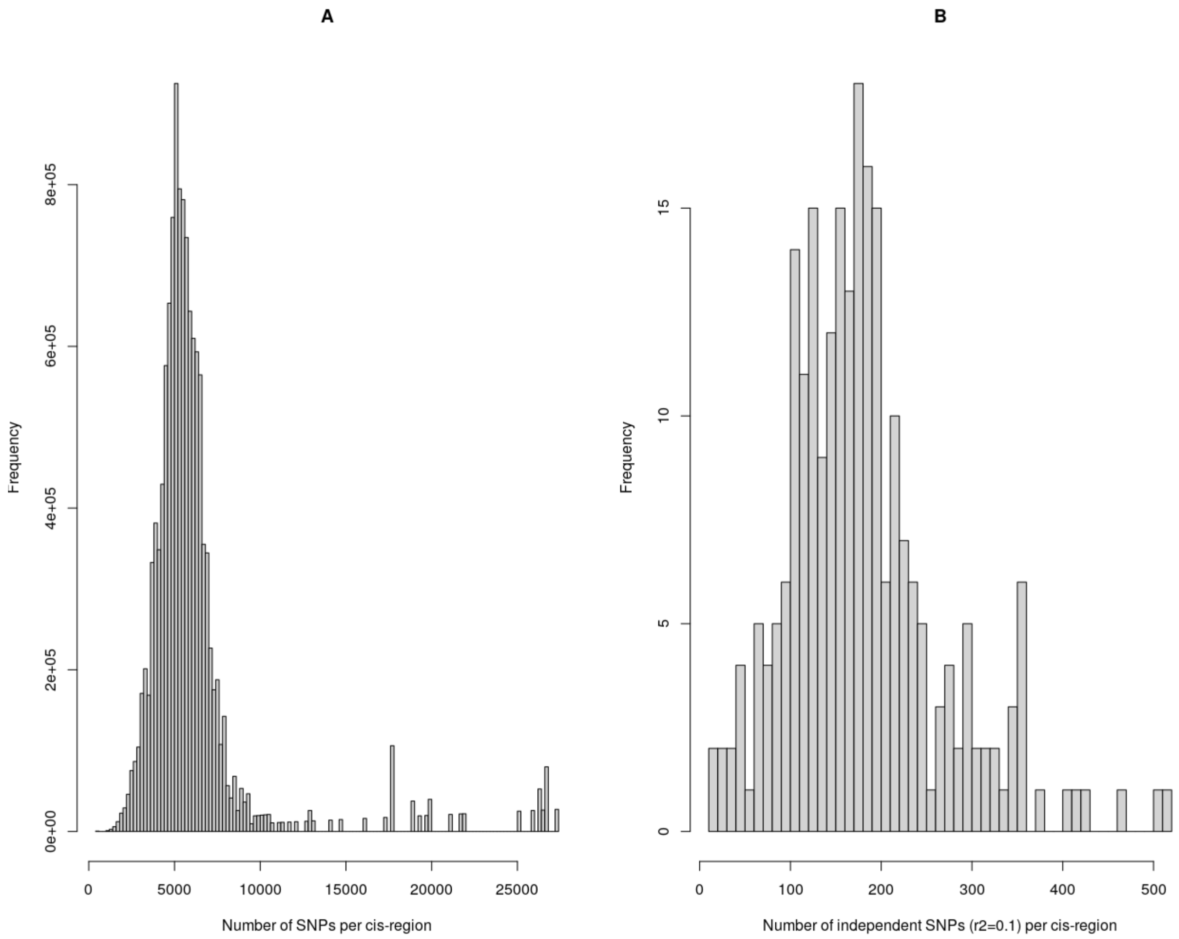

Supplementary figure 5: Histograms of number of tested SNPs per cis-region for all proteins with less than 75% missingness. Panel (a) shows the distribution of total number of SNPs per cis-region with the average number of SNPs equal to 6249.3. Panel (b) shows the distribution of number of independent SNPs (r^2 =0.1) per cis-region with the average number of SNPs equal to 180.4 (min,max 12-511), corresponding to a Bonferroni p-value of 2.77E-04. Across all cis-associations FDR (Q value) estimate at FDR 5% corresponds to a nominal p-value < 5.54E-04, FDR 1% to a nominal p-value < 7.63E-05.

Supplementary figure 6. Comparison of pQTL effect sizes in KARMA with previously published pQTL from the SCALLOP consortium

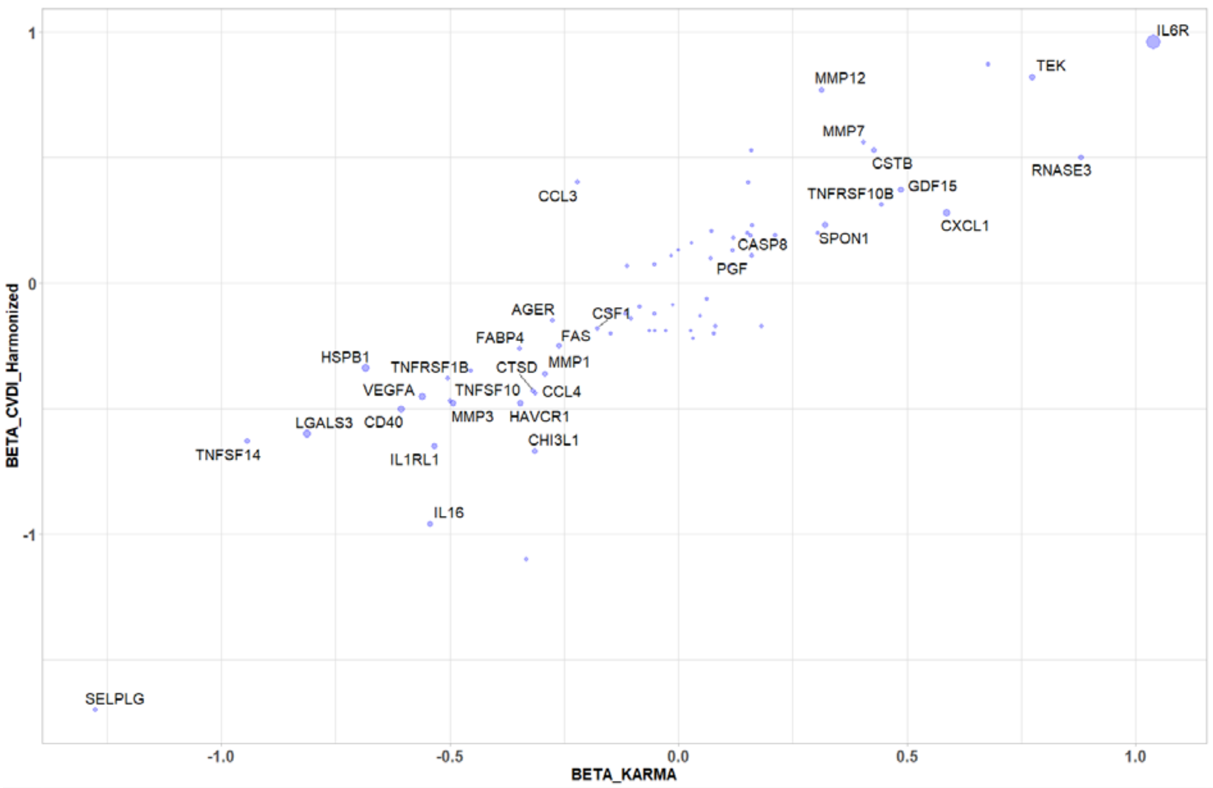

Supplementary figure 6: The beta-estimates for cis-pQTLs identified in the SCALLOP consortium and reported in Folkersen L et. Al. Nat. Metab. 2020 [1] were compared with the beta-estimates of the same cis-pQTL in the KARMA study. The R2 correlation coefficient for the subset of pQTLs with nominal significance in KARMA (p<0.05) was 0.91.

Supplementary figure 7. Mirror plot of CD160 and breast cancer risk

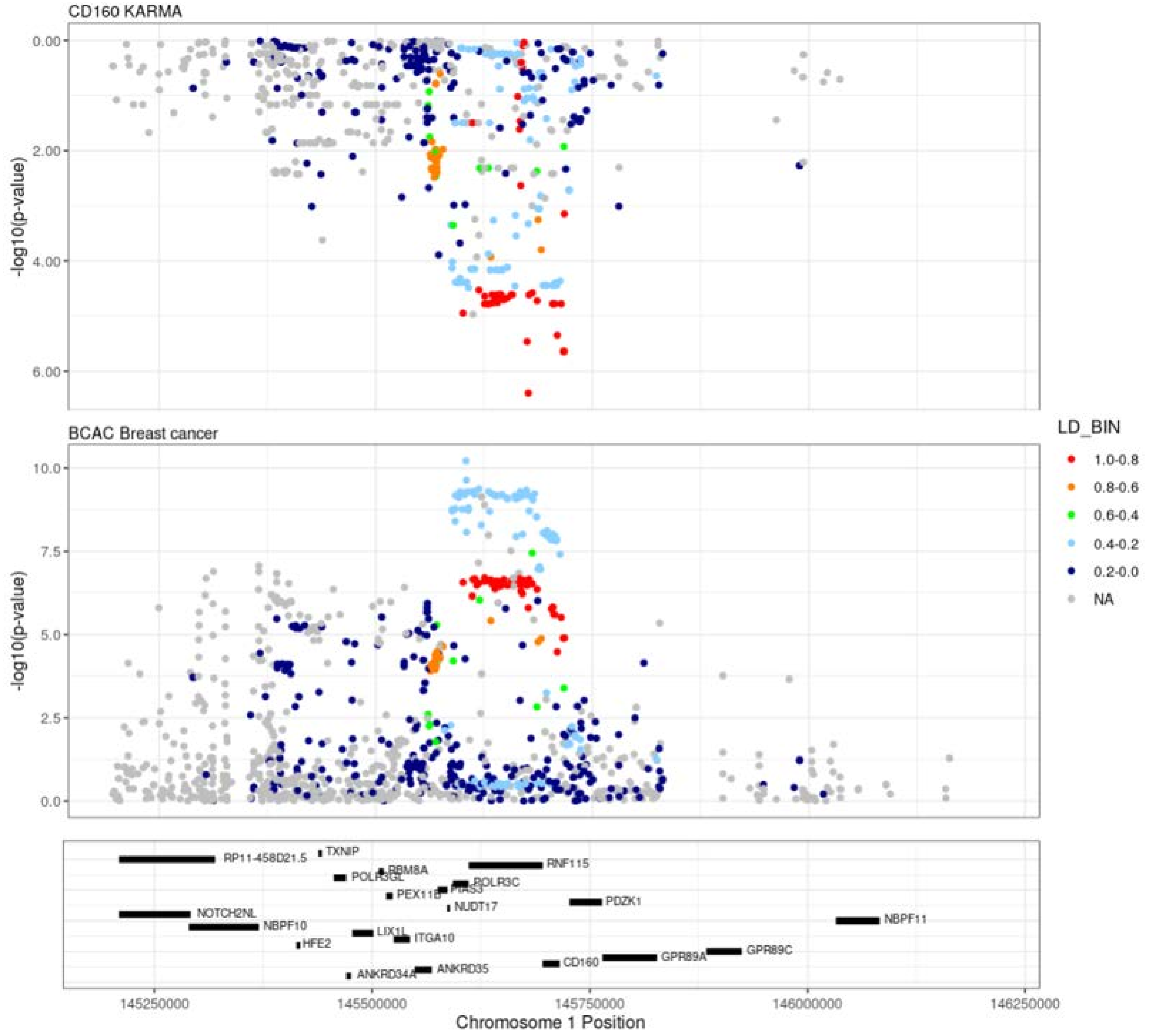

Supplementary figure 7: Mirror plot showing the genomic region of the CD160 cis-pQTL from the KARMA association analysis (top), and the same region in the BCAC breast cancer case/control analysis (bottom). The Y-axis shows the -log(10) p-value, the X-axis shows genomic position in hg19. LD stands for linkage disequilibrium. The LD information shown is based on LD calculations for the lead pQTL identified in the KARMA cohort, with individual variants shown in supplementary table 1, with the same variants highlighted for breast cancer risk in the BCAC data. R package RACER version 1.0.0 (https://github.com/oliviasabik/RACER) were used for the figure.

Supplementary figure 8. Mirror plot of DNPH1 and breast cancer risk

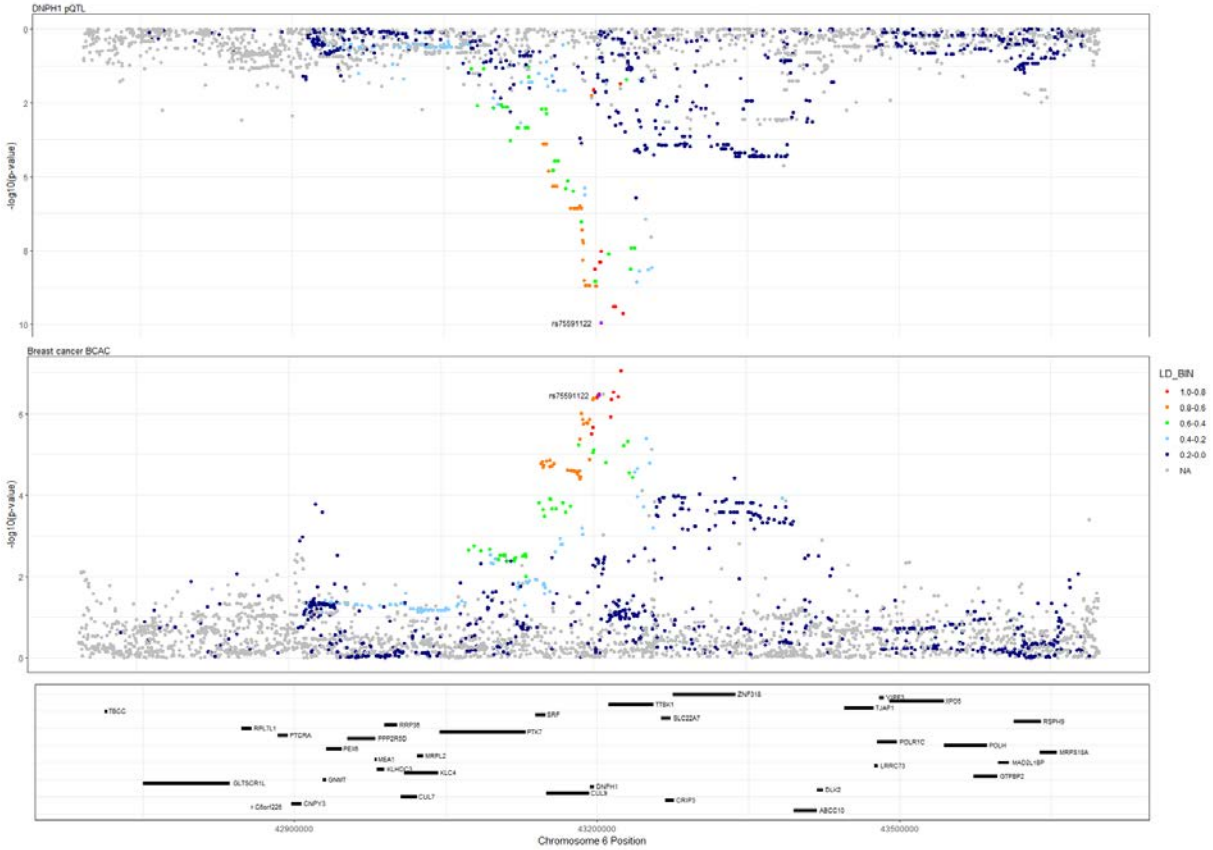

Supplementary figure 8: Mirror plot showing the genomic region of the DNPH1 cis-pQTL from the KARMA association analysis (top), and the same region in the BCAC breast cancer case/control analysis (bottom). The Y-axis shows the -log(10) p-value, the X-axis shows genomic position in hg19. LD stands for linkage disequilibrium. The LD information shown is based on LD calculations for the lead pQTL identified in the KARMA cohort, with individual variants shown in supplementary table 1, with the same variants highlighted for breast cancer risk in the BCAC data. R package RACER version 1.0.0 (https://github.com/oliviasabik/RACER) were used for the figure.

Supplementary figure 9. Mirror plot of LAYN and breast cancer risk

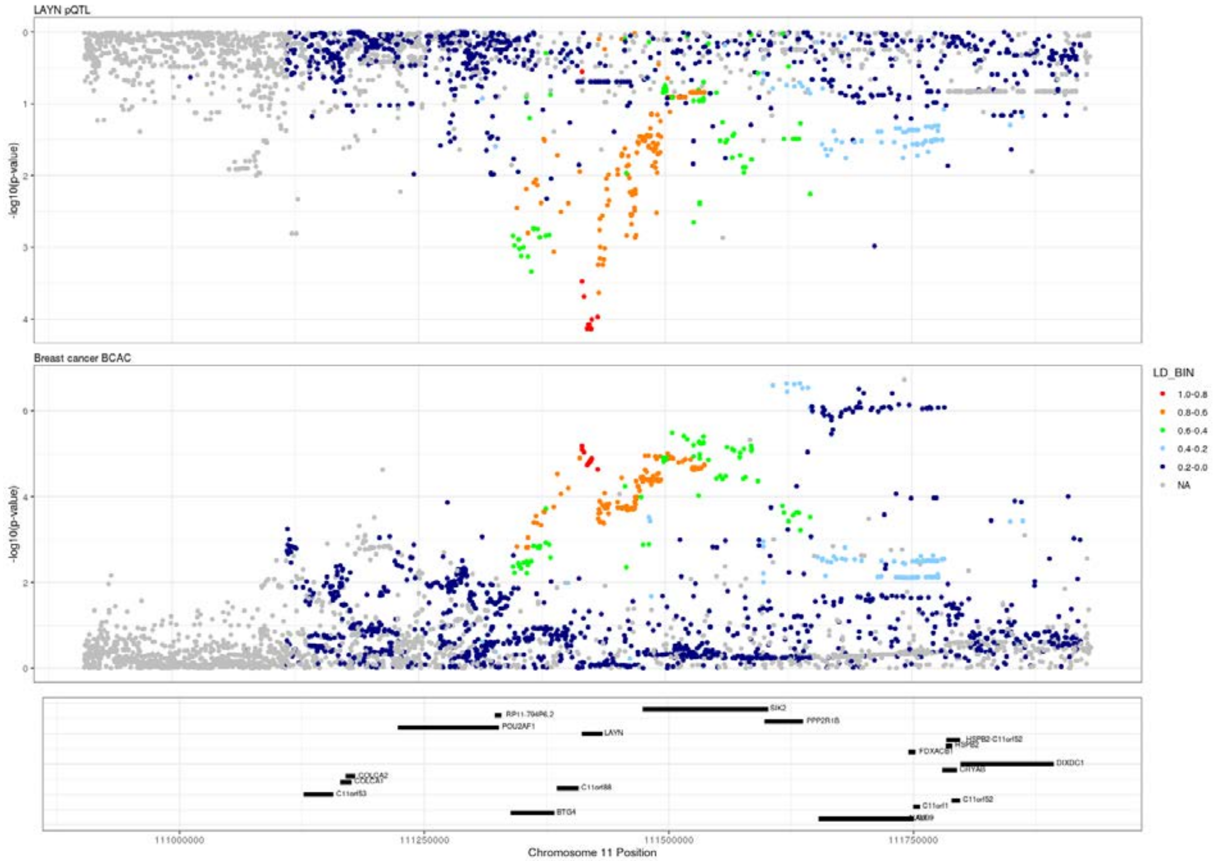

Supplementary figure 9: Mirror plot showing the genomic region of the LAYN cis-pQTL from the KARMA association analysis (top), and the same region in the BCAC breast cancer case/control analysis (bottom). The Y-axis shows the -log(10) p-value, the X-axis shows genomic position in hg19. LD stands for linkage disequilibrium. The LD information shown is based on LD calculations for the lead pQTL identified in the KARMA cohort, with individual variants shown in supplementary table 1, with the same variants highlighted for breast cancer risk in the BCAC data. R package RACER version 1.0.0 (https://github.com/oliviasabik/RACER) were used for the figure.

Supplementary figure 10. Mirror plot of LRRC37A2 and breast cancer risk

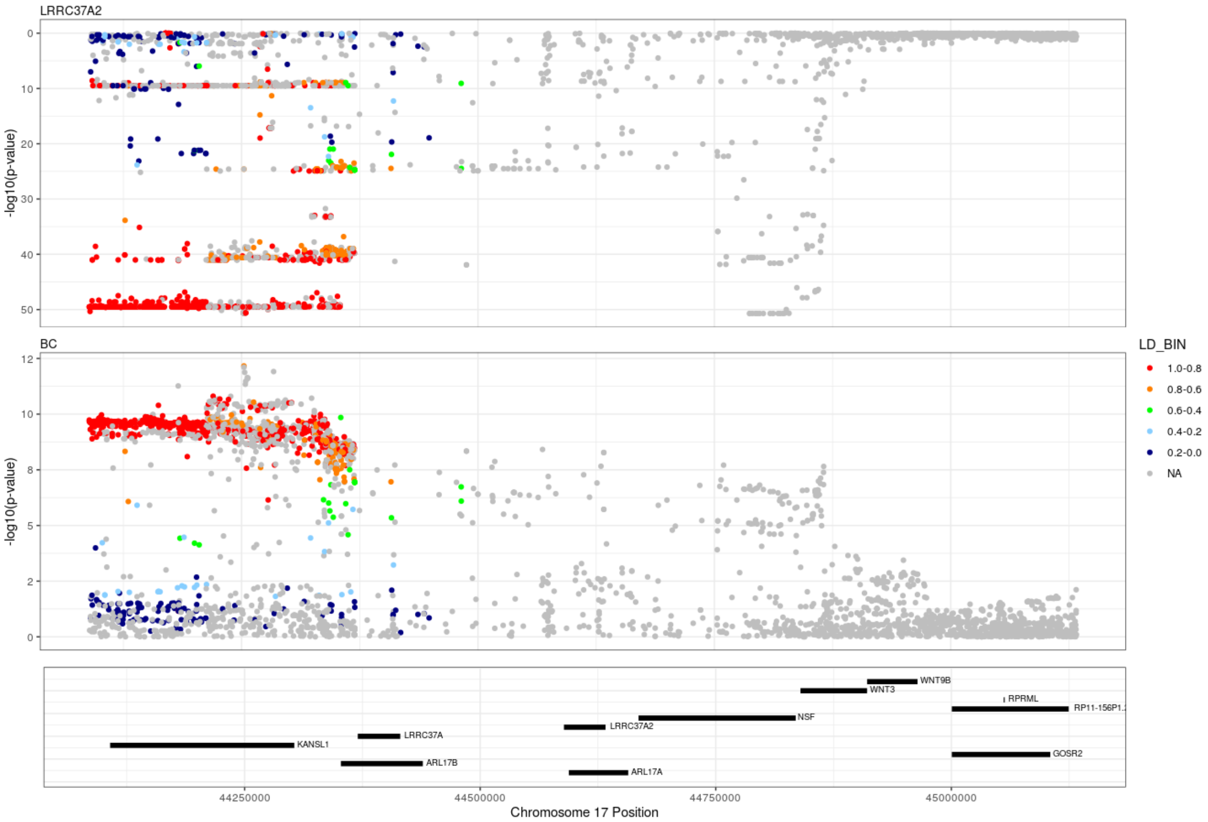

Supplementary figure 10: Mirror plot showing the genomic region of the LRRC37A2 cis-pQTL from the KARMA association analysis (top), and the same region in the BCAC breast cancer case/control analysis (bottom). The Y-axis shows the -log(10) p-value, the X-axis shows genomic position in hg19. LD stands for linkage disequilibrium. The LD information shown is based on LD calculations for the lead pQTL identified in the KARMA cohort, with individual variants shown in supplementary table 1, with the same variants highlighted for breast cancer risk in the BCAC data. R package RACER version 1.0.0 (https://github.com/oliviasabik/RACER) were used for the figure.

Supplementary figure 11. Mirror plot of TLR1 and breast cancer risk

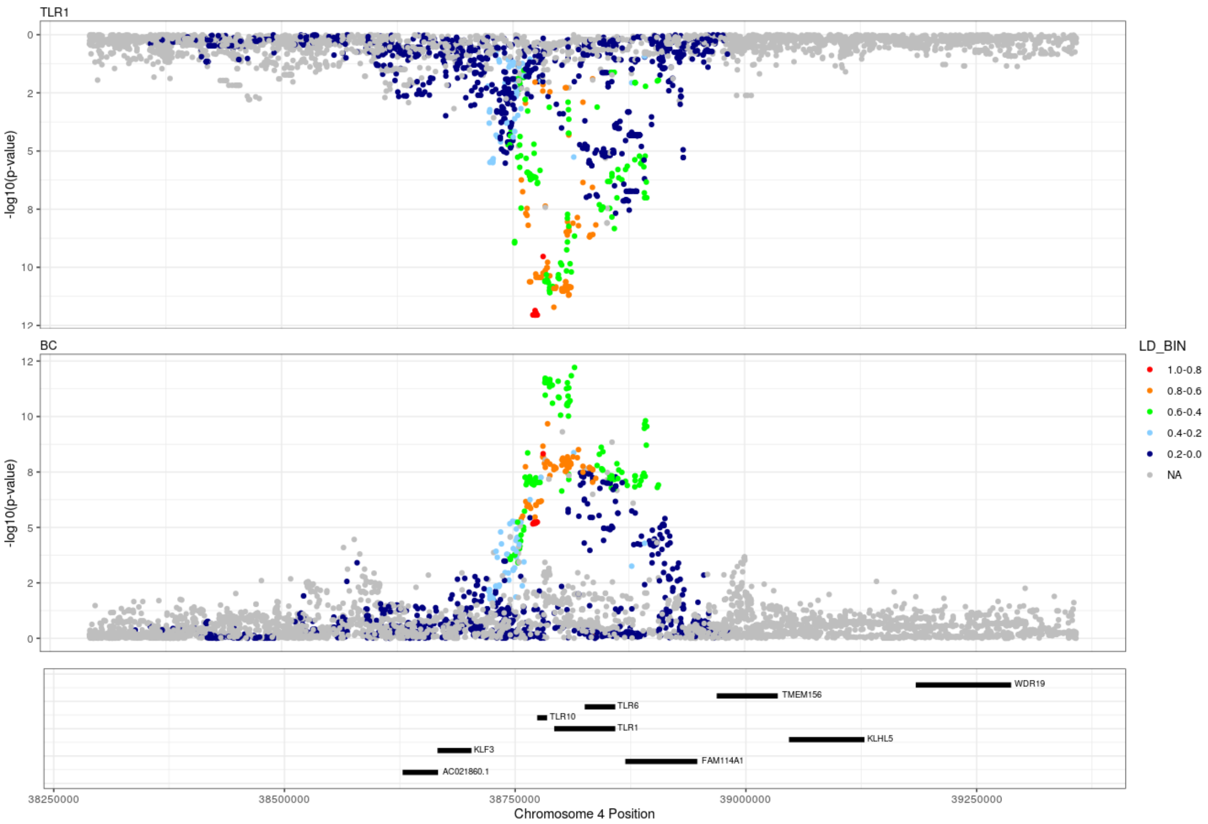

Supplementary figure 11: Mirror plot showing the genomic region of the TLR1 cis-pQTL from the KARMA association analysis (top), and the same region in the BCAC breast cancer case/control analysis (bottom). The Y-axis shows the -log(10) p-value, the X-axis shows genomic position in hg19. LD stands for linkage disequilibrium. The LD information shown is based on LD calculations for the lead pQTL identified in the KARMA cohort, with individual variants shown in supplementary table 1, with the same variants highlighted for breast cancer risk in the BCAC data. R package RACER version 1.0.0 (https://github.com/oliviasabik/RACER) were used for the figure.

Supplementary figure 12. PCA analysis of Olink explore proteins per panel

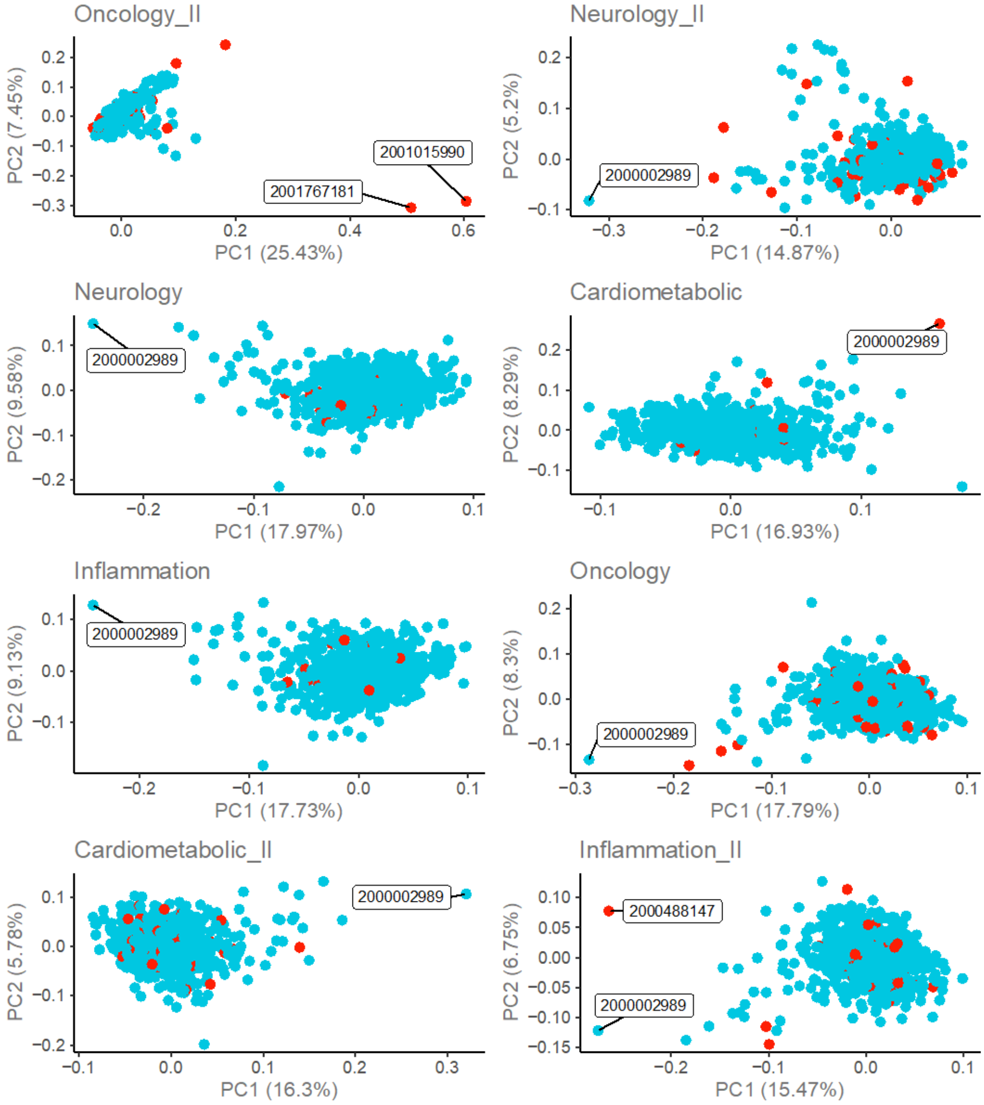

Supplementary figure 12: Principal component (PC) analysis of per-panel Olink Explore data in samples included in the present study. Each dot represents an individual sample.

Supplementary methods, Olink Explore lab protocol

Olink Explore is the latest commercial proteomics product offered by Olink. Explore combines the well-established Proximity Extension Assay technology with Next generation Sequencing offering the possibility of higher multiplexing while still maintaining the specificity and sensitivity that PEA is known for. In addition, using NGS instead of a qPCR based readout method drastically increases throughput.
The PEA technology uses matching pairs of probes consisting of oligonucleotide labelled antibodies that target specific biomarkers with great specificity [2,3]. The PEA probes when bound to their target antigen will produce a binding complex where the complementary oligonucleotides of the two probes exist in close proximity to each other, enabling the formation of a PCR target sequence that is capable of being amplified by the DNA polymerase during PCR. After amplification, the DNA amplicons are detected and quantified in order to give the relative levels of the protein biomarkers present in the sample measured. In other words, the DNA signal that is produced and amplified functionally works as a proxy for the protein levels present in the sample. The dual targeting of probes has been proven to produce outstanding specificity enabling for a high degree of multiplexing while still maintaining sensitivity and a broad dynamic range. PEA was initially developed as a method to analyze proteins that are present in plasma and serum samples, but it is perfectly feasible to utilize this technology in other matrices as well.
The first iteration of Olink Explore panel measured close to 1500 hundred proteins per sample [4]. In the new Explore 3K, this has now expanded to around 3000 proteins [supplementary table 1] per sample. It should be noted that the exact numbers of assays run are not entirely fixed. While most assays in Explore perform well there are a few assays in the low abundant range that are capricious and do not always perform up to standard depending on the probe batch. The performance of the assays is validated by Olink before panel inclusion.
The setup for the 1.5k Explore consists of running four panels in parallel, each panel harboring around approximately one quarter of all assays. Each Explore panel corresponded to one 384-well plate in the Olink protocol. The 3k setup is very similar to the 1.5k setup but instead of assays being split across 4 panels they are split across 8 panels. The first four 1.5k Explore panels were named Cardiometabolic (CAR), Inflammation (INF), Neurology (NEU) and Oncology (ONC), with the 1500 assays being allocated into these four categories based on their relevance as biomarkers. The new assays added for the 3k iteration were divided on a similar basis forming the four new panels Cardiometabolic II (CARII), Inflammation (INFII), Neurology II (NEUII) and Oncology II (ONCII).
The protocol for running the new Explore panels is generally the same as for the four original panels. The one major change is the modified sample preparation part of the protocol. The new panels, just like the original panels, have their assays divided into abundance blocks based on how prevalent a specific protein is in plasma/serum. Given that the dynamic range for proteins in plasma is extraordinarily wide, spanning more than ten orders of magnitude, amplifying from DNA template with these built-in differences will lead to certain low abundant amplicons being outcompeted by amplicons that are massively overrepresented relative to the low abundant assays. The solution to this in the original as well as new panels is to form separate probe pools corresponding to abundance, referred to as abundance blocks, where probes for biomarkers of similar abundance are incubated and amplified together. The abundance blocks are later pooled according to sample, making sure that high and low abundant assays for the same sample are represented in the final library that will eventually be sequenced. All panels are divided into four abundance blocks, Block A-D, each optimized to be compatible with a specific plasma dilution created during sample preparation.

Sample Preparation

The 3k Explore protocol starts with sample preparation, where each sample is split into dilutions, corresponding to one or more blocks throughout the eight Explore 3k panels. Samples as well as the control samples are serially diluted using Olink Sample Dilution buffer, giving rise to five different dilutions: undiluted sample, 1:10, 1:100, 1:1000 and 1:100 000 dilution. The 1:100 000 dilution is a new addition to the sample preparation protocol for Explore 3k, since the 3k iteration of Explore contains a collection of high abundant biomarkers, that require this extra dilution step to ensure that they remain withing the range of their related assay. All dilutions are performed with the help of the Dragonfly Discovery and Mosquito LV liquid handling instruments from SPT Labtech, automating large parts of the workflow as well as allowing for reduction of sample and reagent consumption. Because of this, Explore 3k only requires a total volume of 20 µL from each test-sample in order to run all eight panels.

Sample Incubation

The Incubation mixes, 32 in total, for each block and panel are set up and combined with the samples of appropriate dilution. In this way each sample will be combined with each block ensuring that the samples are tested across all assays yet ensuring that those assays are within appropriate range and will be amplified properly in the first PCR step (PCR1). The samples are incubated at +4°C overnight. The incubation setup is performed by the Mosquito LV liquid handling instruments used in previous steps. Mirroring the original Explore 1.5k protocol, in Explore 3k the Mosquito instrument combines 0.6 µL Incubation mix with 0.2 µL sample ensuring that a minimal amount of plasma or serum sample is consumed for the Explore run.

PCR1 and PCR2

After incubation PCR1 mix is added to all reactions by the Dragonfly Discovery and PCR1 is performed. This step extends and amplifies the DNA target sequence arising from the probe-antigen complex that has formed during the incubation step. After PCR1, the four blocks in each panel are pooled according to sample in a PCR1 pooling plate. For Explore 3k this will result in two 384-well pooling plates, where the original Explore panels are collected in the first plate and the new Explore panels are collected in the second. In practical terms, post-PCR1, the Explore 1.5k workflow is duplicated for the new panels in Explore 3k.

Material from the PCR1 pooling plates is used as template when setting up a new PCR, PCR2, where the pooled PCR1 template, PCR2 master mix and sample indexes are combined. The individual indexes are disseminated so that each sample across all eight panels will be represented by the same index. The index is incorporated into the PCR2 product during amplification. Once PCR2 is completed the PCR2 reactions are pooled panel wise to generate eight library tubes. These steps in the workflow are automated and performed by the Eppendorf's epMotion 5075lc in the standard Olink protocol.

Bead purification and Library quality control

The eight libraries are bead-purified using the AMPure XP magnetic beads purification protocol (Beckman Coulter), removing primers and other DNA fragments that might impede correct NGS readout. Libraries are run on Agilent's 2100 Bioanalyzer using the Agilent High Sensitivity DNA kit for the purpose of quality control.

NGS

The Explore libraries are diluted to the proper concentration according to Olink specification and prepped using the Illumina NovaSeq Xp workflow, enabling the libraries for each panel to be run on separate lanes on the flowcells. Standard procedure at Olink is to run libraries on the Illumina NovaSeq 6000 system. In Explore 3k, custom recipes are used for the NovaSeq 6000 that utilizes dark cycles for common regions. Hence no PhiX needs to be added to the libraries before sequencing, leading to increased library reads. The sequence data is processed and normalized to produce Olinks relative quantification unit Normalized Protein eXpression (NPX)[4]. Briefly, counts of matched sequence reads for each combination of assay and sample barcodes are divided by the counts of extension controls (ExtCtrl) with the same sample barcode. These controls are spiked into every sample at known concentrations in the immunoreaction step. For assay i in sample j, the non-normalized NPX (ExtNPX) is thus defined as:

ExtNPXi,j=log2 (Counts(SamplejAssayi)Counts(ExtCtrlj))ExtNPXi,j=log2 CountsSamplejAssayiCountsExtCtrlj

The plate control sample, consisting of pooled plasma run in triplicate on each plate, is used to correct for variation between plates. This is done by subtracting the median of the plate control ExtNPX per assay and plate:

NPXi,j,k=ExtNPXi,j,k−median(ExtNPXplateCtrls i,k)NPXi,j,k=ExtNPXi,j,k−medianExtNPXplateCtrls i,k

where k = plate. To further minimize potential technical variation across plates, an additional normalization step can be performed by subtracting the median NPX value per assay and plate:

NPXIntNorm i,j,k=NPXi,j,k−median(NPXi,k)NPXIntNorm i,j,k=NPXi,j,k−medianNPXi,k

This step is referred to as intensity normalization and is only recommended when samples are adequately randomized over plates.
The additional internal and external controls included in every run are used to monitor technical consistency and quality according to Olinks standard criteria [4].

Quality Control
The Olink QC-system includes negative controls, used to monitor the background noise and to set the limit of detection (LOD). Supplementary figure 1 and Supplementary table 1 show the percentage of samples with NPX above LOD.
A principal component analysis of all data was performed to detect outliers and to inform potential sample exclusions. Of the 598 samples that were included in the analysis, one sample was excluded entirely, two samples were excluded from analysis of Explore ONC-II and two samples were excluded from analysis of the Explore INF-II panel data (Supplementary figure 11).

Supplementary References

1. Folkersen, L. *et al.* Genomic and drug target evaluation of 90 cardiovascular proteins in 30,931 individuals. *Nat Metab* **2**, 1135-1148 (2020).

2. Assarsson, E. *et al.* Homogenous 96-plex PEA immunoassay exhibiting high sensitivity, specificity, and excellent scalability. *PLoS One* **9**, e95192 (2014).

3. Lundberg, M., Eriksson, A., Tran, B., Assarsson, E. & Fredriksson, S. Homogeneous antibody-based proximity extension assays provide sensitive and specific detection of low-abundant proteins in human blood. *Nucleic Acids Res* **39**, e102 (2011).

4. Wik, L. *et al.* Proximity Extension Assay in Combination with Next-Generation Sequencing for High-throughput Proteome-wide Analysis. *Mol Cell Proteomics* **20**, 100168 (2021).
