# Supplementary material for: Evaluation of circulating plasma proteins in breast cancer using Mendelian randomisation
Source: Nat Commun. 2023 Nov 24;14:7680. doi: 10.1038/s41467-023-43485-8 (PMC10667261; doi:10.1038/s41467-023-43485-8)
Supplement: Supplementary file 3 — Description of Additional Supplementary Files Document [file 41467_2023_43485_MOESM3_ESM.pdf]

Description of Additional Supplementary Files

Supplementary Data 1- Proteins measured in KARMA on the Olink Explore I and II panels and % detected above limit of detection

Supplementary Data 2- Comparisons with human protein atlas blood data for proteins also measured in the present study

Supplementary Data 3- Association between Olink proteins and clinical characteristics of the KARMA women

Supplementary Data 4- cis-pQTL identified in the KARMA study.

Supplementary Data 5- Mendelian randomization results for breast cancer, using BCAC as outcome data

Supplementary Data 6- Mendelian randomization results for breast cancer risk factors
