# Supplementary material for: Evaluation of circulating plasma proteins in breast cancer using Mendelian randomisation
Source: Nat Commun. 2023 Nov 24;14:7680. doi: 10.1038/s41467-023-43485-8 (PMC10667261; doi:10.1038/s41467-023-43485-8)
Supplement: Supplementary file 5 — Reporting Summary [file 41467_2023_43485_MOESM5_ESM.pdf]

nature portfolio

Corresponding author(s): Anders Malarstig

Last updated by author(s): Oct 6, 2023

Reporting Summary

Nature Portfolio wishes to improve the reproducibility of the work that we publish. This form provides structure for consistency and transparency in reporting. For further information on Nature Portfolio policies, see our Editorial Policies and the Editorial Policy Checklist.

Statistics

For all statistical analyses, confirm that the following items are present in the figure legend, table legend, main text, or Methods section.

n/a	Confirmed	
☐	☒	The exact sample size (n) for each experimental group/condition, given as a discrete number and unit of measurement
☐	☒	A statement on whether measurements were taken from distinct samples or whether the same sample was measured repeatedly
☐	☒	The statistical test(s) used AND whether they are one- or two-sided<br>*Only common tests should be described solely by name; describe more complex techniques in the Methods section.*
☐	☒	A description of all covariates tested
☐	☒	A description of any assumptions or corrections, such as tests of normality and adjustment for multiple comparisons
☐	☒	A full description of the statistical parameters including central tendency (e.g. means) or other basic estimates (e.g. regression coefficient) AND variation (e.g. standard deviation) or associated estimates of uncertainty (e.g. confidence intervals)
☐	☒	For null hypothesis testing, the test statistic (e.g. F, t, r) with confidence intervals, effect sizes, degrees of freedom and P value noted<br>*Give P values as exact values whenever suitable.*
☒	☐	For Bayesian analysis, information on the choice of priors and Markov chain Monte Carlo settings
☒	☐	For hierarchical and complex designs, identification of the appropriate level for tests and full reporting of outcomes
☐	☒	Estimates of effect sizes (e.g. Cohen's d, Pearson's r), indicating how they were calculated

Our web collection on statistics for biologists contains articles on many of the points above.

Software and code

Policy information about availability of computer code

Data collection	The KARMA database is kept in SAS -a proprietary software developed by the SAS Institute. Clinical data from the KARMA database are exported to R for statistical analysis.
Data analysis	PLINK 2.0 is an established publicly available software for genetic association analysis. All other statistical analyses were conducted using R, which is an open-source software. The scripts and pipelines for quality control, association analyses and Mendelian randomisation are available at https://github.com/Schwenk-Lab/KARMA_pQTL_MR and https://github.com/Olink-Proteomics/KARMA_MR_NC.

For manuscripts utilizing custom algorithms or software that are central to the research but not yet described in published literature, software must be made available to editors and reviewers. We strongly encourage code deposition in a community repository (e.g. GitHub). See the Nature Portfolio guidelines for submitting code & software for further information.

Data

Policy information about availability of data

All manuscripts must include a data availability statement. This statement should provide the following information, where applicable:

- Accession codes, unique identifiers, or web links for publicly available datasets
- A description of any restrictions on data availability
- For clinical datasets or third party data, please ensure that the statement adheres to our policy

The cis-pQTL GWAS summary-level data generated in this study have been deposited in the Zenodo data repository under accession code doi: 10.5281/zenodo.8387905, with the URL https://zenodo.org/record/8387905. For previously published GWAS studies on breast cancer risk and risk factors, the summary-

level data are available at https://bcac.ccge.medschl.cam.ac.uk/bcacdata/ or at the MRC IEU OpenGWAS database [https://gwas.mrcieu.ac.uk/] [https://gwas.mrcieu.ac.uk/datasets/ieu-a-1126/], ieu-a-1127, ieu-a-1128. Access to FinnGen data are available for request at https://www.finngen.fi/en/access_results whereas access to UK-biobank genotypes and breast cancer case-control data can be accessed at https://www.ukbiobank.ac.uk/.
The process for accessing raw data i.e. phenotypes, biospecimen and genotypes from the KARMA study is described here https://karmastudy.org/contact/data-access/.

Human research participants

Policy information about studies involving human research participants and Sex and Gender in Research.

Reporting on sex and gender	This study involves female participants only. Breast cancer may occur also in males but less than 1 % of breast cancers diagnosed occur in males.
Population characteristics	All women from age 40 in Sweden are invited to participate in the mammography screening program, which is aimed at early detection of breast cancer to decrease the societal and personal burden of the disease. In general, women are seen every second year. The participants in the KARMA study were recruited at 4 different clinics providing mammography screening. Further details are given here https://karmastudy.org/
Recruitment	All women participating in the Swedish mammography screening program were invited to participate. About 80% of eligible women attend screening regularly, which is among the highest recorded in the world, which makes the KARMA cohort generally representative of the general population.
Ethics oversight	The study was approved by the Stockholm ethical review board, https://etikprovningsmyndigheten.se/en/.

Note that full information on the approval of the study protocol must also be provided in the manuscript.

Field-specific reporting

Please select the one below that is the best fit for your research. If you are not sure, read the appropriate sections before making your selection.

[x] Life sciences [ ] Behavioural & social sciences [ ] Ecological, evolutionary & environmental sciences

For a reference copy of the document with all sections, see nature.com/documents/nr-reporting-summary-flat.pdf

Life sciences study design

All studies must disclose on these points even when the disclosure is negative.

Sample size	We selected all KARMA samples from women who were a) diagnosed with breast cancer within 2 years from the mammography screen b) were recruited from the Southern Sweden mammography clinics. This number was n=299. We also selected random controls from the same mammography clinics to minimise differences in pre-analytical handling of samples. Given the exploratory nature of the study, a statistical power calculation was not considered to be helpful and was therefore not performed. However, we note that for discovery of pQTL, an effect size of 0.25 standard deviations of protein level per allele would be readily detected in the sample size of n=598.
Data exclusions	The KARMA genotyping was performed on standard Illumina panels, iSelect or Omniexpress followed by imputation and standard QC filtering such as removing variants that deviate from Hardy-Weinberg equilibrium and those with minor allele frequencies below 1%. Qualtiy control was also applied to the Olink proteomics analyses according to predefined criteria, described here https://pubmed.ncbi.nlm.nih.gov/34715355/
Replication	We performed two independent replication attempts of the pQTL discovered in our KARMA study. One third of the cis-pQTL that were previously reported by Folkersen et al in Nat Metab. 2020 were replicated, despite considerable smaller sample size in KARMA. We further replicated a subset of pQTL for 374 proteins reported by Pietzner et al. Nat Comm 2021 for the overlapping set of 569 proteins In addition, we performed replication analysis of the main results reported. Of the 7 proteins that reached statistical significant in the discovery analysis, using data from the breast cancer association consortium, 5 were replicated in the independent FinnGen plus UK-biobank breast cancer data.
Randomization	All incident cases from Southern Sweden were included in this study and was therefore not a random selection. For the breast-cancer free control samples we used the Matchit function implemented in R to randomly draw controls so that the median age at blood draw in cases and controls was similar (median matching)". For the Olink measurements, cases and controls were randomised across the analyses plates in a manner that was blinded to the cases control samples to avoid batch effects.
Blinding	The lab personell and study team was blinded to the breast cancer case-control status. The status was unblinded when matching the Olink protein measurements to the appropriate KARMA study number, and analysis was conducted.

Reporting for specific materials, systems and methods

We require information from authors about some types of materials, experimental systems and methods used in many studies. Here, indicate whether each material, system or method listed is relevant to your study. If you are not sure if a list item applies to your research, read the appropriate section before selecting a response.

Materials & experimental systems

n/a	Involved in the study
☒	☐ Antibodies
☒	☐ Eukaryotic cell lines
☒	☐ Palaeontology and archaeology
☒	☐ Animals and other organisms
☒	☐ Clinical data
☒	☐ Dual use research of concern

Methods

n/a	Involved in the study
☒	☐ ChIP-seq
☒	☐ Flow cytometry
☒	☐ MRI-based neuroimaging
